# Editorial: Critical data and algorithm studies

**DOI:** 10.3389/fdata.2023.1193412

**Published:** 2023-05-10

**Authors:** Katja Mayer, Jürgen Pfeffer

**Affiliations:** ^1^Science and Technology Studies, University of Vienna, Vienna, Austria; ^2^Computational Social Science and Big Data, Technical University of Munich, Munich, Germany

**Keywords:** critical data studies, critical data science, critical algorithm studies, data work, social theory, big data, computational social science

## 1. Introduction

As digitalisation, mobile computing and social media platforms have become ubiquitous in modern life, there has been a surge in the use of big data analytics and data-driven research approaches. Scholars in human-computer interaction, critical data studies, and critical algorithm studies have long been concerned with the social challenges posed by data science, including issues of bias, opaque access to data and infrastructure, and issues of representation (Agre, 1997; Boyd and Crawford, 2012; Kitchin, 2014; Zook et al., 2017; Moats and Seaver, 2019). To bridge the gap between cultures of critique and those of practice, this Research Topic is dedicated to bringing together critical expertise from data-driven research fields to reflect on the social impact of their research. Furthermore, the papers in this Research Topic address a range of issues, from understanding the limitations of social data and methods, the need to consider social theories for better outcomes and research integrity in the datafication of social behavior, to pressing issues of research infrastructures and the complexities of social media research.

## 2. The Research Topic of articles

The first paper in our Research Topic, “*Social data: biases, methodological pitfalls, and ethical boundaries*” by Olteanu et al., lists and discusses the biases and inaccuracies in social data, as well as methodological limitations and ethical concerns. The article argues for the importance of auditing social data and algorithms to address potential biases and makes four recommendations: detailed documentation of datasets and models; expanding social data research to different platforms, topics, timings, and subpopulations; enabling transparency mechanisms to facilitate auditing of social software; and broadening research on guidelines, standards, methodologies, and protocols to address the limitations of social data.

In this vein, Radford and Joseph's paper argues that social theory is crucial to addressing problems with machine learning models used to analyse social data. Technical solutions alone are not enough. Social theory provides insights into methodological and interpretive questions that cannot be answered by technical fixes. The paper recommends further research into how social theory can be applied to address bias and inequality in machine learning models. By developing a systematic theoretical framework for social data, researchers can identify and address various challenges and limitations associated with the use of social data, such as biases in data collection and processing, methodological limitations, and ethical concerns. A structured approach to social data analysis can lead to more accurate and reliable results, improving decision-making and policy development.

The paper by Poechhacker and Kacianka applies a theoretical perspective to the ongoing debate on algorithmic accountability in automated decision making and machine learning. It focuses on the use of structural causal models (SCMs) as a means of establishing accountability by describing the causal relationships between different factors in an algorithmic system. As such, SCMs provide transparency that allow for public scrutiny, as people can review the rules and decisions to ensure that they are fair and ethical. However, the authors argue that the concept of causality needs further exploration. They bring insights from social theory, particularly pragmatism, and suggest that formal expressions of causality need to be considered within the social system in which they are applied.

In “*Decentralized but globally coordinated biodiversity data*” by Sterner et al., the authors draw our attention to the central role of research infrastructures and their governance. They argue that centralized biodiversity data aggregation is failing to meet societal needs due to data quality shortcomings and propose a decentralized approach to data coordination. The authors suggest that this approach will lead to sustained expert engagement, higher quality data products and greater societal impact. The decentralized approach encourages the emergence and evolution of multiple self-identifying communities of practice that can control the social and informational design of their local data infrastructures.

The paper “*The datafication of hate: expectations and challenges in automated hate speech monitoring*” by Laaksonen et al. reflects on an action research setting aimed at monitoring social media updates for hate speech during the Finnish local elections in 2017. The study examines how hate speech emerged as a technical problem, and how an algorithmic solution was developed using supervised machine learning. The paper highlights the oversimplification of the automated approach and research design, and suggests practical implications for hate speech detection.

The need to reflect on one's own research and data handling is also considered in the paper by Kinder-Kurlanda and Weller. The authors argue that the details of scientific practice in data science and computational social science are not well-described in the literature. They propose a perspective that recognizes the everyday “data work” required to conduct social media research at different stages of a data lifecycle. The authors highlight the complexity faced by social media researchers and suggest that documenting research decisions is necessary to better understand what drives social media research and to address structural challenges in the research ecosystem. The overall outcome of such documentation is improved research rigor and transparency, leading to more accurate and trustworthy results.

The paper by Allhutter et al. examines the societal impact of algorithms in decision making. They discuss the algorithmic profiling used by the Austrian Public Employment Service (AMS) to categorize job seekers based on their prospects in the labor market. Drawing on their interdisciplinary collaboration, the authors highlight the tensions, challenges and biases inherent in the AMS algorithm and question the objectivity and neutrality of data claims and evidence-based decision making. The paper thus sheds light on (semi)automated management practices in employment agencies and the framing of unemployment under austerity policies, providing insights not only for critical data studies but also for public service policies.

In “*Staying with the trouble of networks*,” van Geenen et al. examine the use of network visualizations in different fields, such as scientific research or journalism, and argue that problems with such visualization practices can provide opportunities to reflect on knowledge practices in social contexts. While network visualizations are both discovery and narrative tools, the authors make explicit the epistemic assumptions built into network tools and emphasize the need to pay attention to the cultural practices of interpreting and understanding social relations with network analysis. By attending to the different settings and situations in which network graphs and maps are created and used, the authors suggest that we can develop a more nuanced understanding of the role of networks in collective forms of inquiry and sense-making. The article contributes to the concept of critical data practice by highlighting the need to consider the social and ethical implications of data.

## 3. Conclusion

In summary, this Research Topic has brought together a diverse range of articles that highlight this need for critical technical practice in data-driven research areas. The articles contribute to the growing fields of Critical Data Studies and Critical Algorithm Studies by addressing scientific, ethical, and social challenges, and by promoting a cross- and transdisciplinary exchange between practical and critical positions, thus encouraging collaborations between computer scientists, social scientists, humanities scholars, journalists, policy makers, and activists, and providing a productive intersection between cultures of critique and those of practice. Overall, the Research Topic offers a valuable contribution to ongoing critical reflection on scientific methods, data sources, modeling, validation, replication and review procedures, while highlighting the performative and normative aspects of data science practices.

## Author contributions

All authors listed have made a substantial, direct, and intellectual contribution to the work and approved it for publication.
